# COVID-19 Pandemic Effects on Cervical Cancer Diagnosis and Management: A Population-Based Study in Romania

**DOI:** 10.3390/diagnostics12040907

**Published:** 2022-04-06

**Authors:** Alin Popescu, Marius Craina, Stelian Pantea, Catalin Pirvu, Veronica Daniela Chiriac, Iosif Marincu, Felix Bratosin, Iulia Bogdan, Samer Hosin, Cosmin Citu, Elena Bernad, Radu Neamtu, Catalin Dumitru, Adelina Geanina Mocanu, Claudiu Avram, Adrian Gluhovschi

**Affiliations:** 1 Department of Obstetrics and Gynecology, “Victor Babes” University of Medicine and Pharmacy, 300041 Timisoara, Romania; alinp22@yahoo.com (A.P.); mariuscraina@hotmail.com (M.C.); chiriac.veronica@umft.ro (V.D.C.); citu.ioan@umft.ro (C.C.); ebernad@yahoo.com (E.B.); radu.neamtu@umft.ro (R.N.); dumcatal@yahoo.com (C.D.); adelinamocanu2955@yahoo.com (A.G.M.); adigluhovschi@yahoo.com (A.G.); 2 Department of General Surgery, “Victor Babes” University of Medicine and Pharmacy, 300041 Timisoara, Romania; pirvu.catalin.alexandru@gmail.com; 3 Methodological and Infectious Diseases Research Center, Department of Infectious Diseases, “Victor Babes” University of Medicine and Pharmacy, 300041 Timisoara, Romania; imarincu@umft.ro (I.M.); felix.bratosin7@gmail.com (F.B.); iulia.georgianabogdan@gmail.com (I.B.); 4 Department of Orthopedics, “Victor Babes” University of Medicine and Pharmacy, 300041 Timisoara, Romania; samerhosin@gmail.com; 5 Doctoral School, “Victor Babes” University of Medicine and Pharmacy, 300041 Timisoara, Romania; clauavra@yahoo.com

**Keywords:** SARS-CoV-2, COVID-19, cervical cancer, cancer diagnosis

## Abstract

The Pap test plays a significant role worldwide in the early diagnosis of and high curability rates for cervical cancer. However, the coronavirus disease 2019 (COVID-19) pandemic necessitated the use of multiple drastic measures to stop the spread of the severe acute respiratory syndrome coronavirus 2 (SARS-CoV-2) virus, limiting women’s access to essential invasive and non-invasive investigations for cervical cancer diagnosis. Therefore, we aimed to determine the impact the COVID-19 pandemic had on cancer diagnosis and management in western Romania. A retrospective study design allowed us to compare the last 24 months of the pre-pandemic period with the first 24 months of the COVID-19 pandemic to determine the change in volume of cervical screening tests, the number of newly diagnosed cases and their severity, and the access to cancer care. A drastic 75.5% decrease in the volume of tests was observed in April 2020 during the first lockdown, after which the volume of cases decreased by up to 36.1% in December 2021. The total volume loss of tests during the first 24 months of the pandemic was 49.9%. The percentage of late-stage cervical cancers (III–IV) rose by 17%, while the number of newly diagnosed cancers in our outpatient clinic was significantly lower than the baseline, with a 45% drop. The access to cancer care was negatively influenced, with 9.2% more patients waiting longer to receive test results over four weeks, while taking longer to seek cancer care after diagnosis (6.4 months vs. 4.1 months pre-pandemic) and missing significantly more appointments. The COVID-19 pandemic had a significantly negative impact on cervical cancer diagnosis and management during the first 24 months compared with the same period before the pandemic. Although the numbers are now recovering, there is still a big gap, meaning that many cervical cancer cases were potentially missed. We recommend further interventions to reduce the gap between the pre-pandemic and pandemic period.

## 1. Introduction

Cervical cancer currently occupies second place as the most common cancer in young women between 15 and 44 years old. It is responsible for more than 500 thousand cases every year worldwide [1], ranking as the third most common cancer type [2]. Globally, around 200 thousand women die every year from cervical cancer, and both the mortality rates and incidence rates are highest in developing sub-Saharan countries [3]. Women in the 35–66-years-old age group account for two-thirds of all cervical cancers, with a median age at diagnosis of 49 years [4]. The prognosis in developed countries such as the UK is excellent, where more than 60% of women survive ten years after diagnosis. The 5-year relative survival for the International Federation of Obstetrics and Gynecology (FIGO) stage I is 96% in the United Kingdom and 92% in the United States of America [5]. On the other side, 87% of all deaths caused by cervical cancer happen in developing countries [3].

Intensive screening for cervical cancer allows for early detection by performing the widely used Pap smear with microscopic evaluation. The most frequently observed cancer histology is squamous-cell carcinoma (SCC), accounting for 90% of all cervical cancers, while the remaining part is caused by adenocarcinomas (ADC) in around 9% of cases and other histology variants in 1% [6]. The sexually transmitted human papillomavirus (HPV) is the main cause of the development of SCC of the cervix [7,8]; it is classified into low-risk and high-risk types, with the latter also known as oncogenic HPV type. If the viral infection is not cleared within 12 to 24 months, it will tend to progress to a precancerous state [9], starting as cervical intraepithelial neoplasia (CIN) and evolving to invasive cancer through biochemical and molecular alterations, such as down-regulating e-cadherins [10,11], histidine phosphatase [12], the nucleotide-binding oligomerization domain protein-1 (NOD1) [13], and other proteins. According to the VIVIANE study [14], the highest-risk strains are, in descending order, HPV33, HPV16, HPV18, HPV31, and HPV45. High-risk HPV is found in 99.7% of cervical cancers [15], but other risk factors such as chlamydia trachomatis have been discovered in 40% of SCC cases [16], while the human immunodeficiency virus (HIV) and herpes simplex virus 2 (HSV-2) infections are also associated with cervical cancer [17]. Nutritional behavior, use of oral contraceptive pills, smoking, obesity, and inflammatory diseases are also correlated with a higher chance of developing cervical cancer [18].

Since the start of the coronavirus disease 2019 (COVID-19) pandemic, health services have made steps to expand their admission capacity for COVID-19 patients while decreasing the circulation of non-COVID-19 patients. This objective has been accomplished by minimizing outpatient visits and deferring tests, treatments, and elective surgery. These measures may disproportionately impact cancer patients, since the period between diagnosis and treatment commencement might negatively affect results. National screening programs were temporarily discontinued in certain areas to relieve pressure on health systems by urging the deferral of non-emergency visits, examinations, and treatments during quarantine [19]. Additionally, the medical management of new cases was delayed due to the reorganization of health facilities and patients’ fear of interaction with COVID-19 [20]. In countries such as the United States, a considerable decline in the frequency of screening tests and, subsequently, in the number of malignant and precursor lesions diagnosed has already been documented. In a conservative analysis of the effect of COVID-19 on breast and colorectal cancer screening and treatment in the United States, it is estimated that nearly 10,000 additional deaths will occur over the next decade, representing an increase of approximately 1% in the total number of deaths during a period when we would expect nearly 1 million deaths from the two most common tumors affecting women [21].

Similarly to other European Union countries, the Romanian national screening programs were temporarily put on hold during the lockdown at the pandemic’s peak as a governmental measure to prevent SARS-CoV-2 infection. This program allowed women aged 21 to 65 years old to benefit from free annual Pap smears for the early detection of cervical lesions [22]. Although official statistics regarding the cases of cancer diagnosed during the COVID-19 pandemic have not yet been released, we observed an important decrease in the number of newly diagnosed cases of cervical cancer, as well as a decrease in women requesting investigations for cervical cancer in our clinical practice during the past 24 months of the ongoing pandemic. This raised concerns about the number of cases of cervical cancer that had been missed. Considering this hypothesis, we developed a study that aimed to determine the influence of the first 24 months of the COVID-19 pandemic on the delivery of cervical cancer tests, the stage at which new cervical cancer patients were diagnosed, and their access to cancer care.

## 2. Materials and Methods

An observational study was organized at the University Clinic of Obstetrics and Gynecology “Bega” of the Timis County Emergency Clinical Hospital “Pius Brinzeu” from Timisoara, Romania, affiliated with the “Victor Babes” University of Medicine and Pharmacy. The research population and characteristics of interest were identified using a population-based administrative database on patients that presented during the study period in the outpatient setting of the same clinic. Our centralized database included patient medical records kept under privacy protection and patient consent, including their medical history, cervical cytology tests, and surgical and oncological data.

In Timis County, a region served by our clinic, approximately 300 thousand women are eligible to undergo cervical cytology tests annually. Women that are diagnosed with a high-grade cytology result following our analysis are advised to undergo a colposcopy, while those with a low-grade cytology result are advised to repeat cytology in six months. Colposcopy enables a doctor to detect a cervical lesion, confirm the diagnosis by biopsy, and recommend therapy for cervical cancer precursors or conservative care in the absence of cancer precursors. The research included all women aged 21 to 65 years old who had cervical screening cytology, colposcopy, or cancer therapy between January 2018 and January 2022. Individuals under the age of 21 were omitted, as were those 65 years and older.

The variables considered for evaluation comprised patient background data; the number of cervical cytology tests, HPV tests, and colposcopies performed; tumor staging; the interval from biopsy to first cancer center visit; and cancer therapy between the pre-pandemic and pandemic period. Cervical cancer staging was based on the 2018 International Federation of Gynecology and Obstetrics (FIGO) staging system [23]. The pre-pandemic period was considered as the 24 months period from January 2018 until January 2020, while the pandemic period comprised the time frame between January 2020 and January 2022.

The Local Commission of Ethics for Scientific Research from the Timis County Emergency Clinical Hospital “Pius Brinzeu” from Timisoara, Romania, operates under article 167 provisions of Law no. 95/2006, art. 28, chapter VIII of order 904/2006; with EU GCP Directives 2005/28/EC, International Conference of Harmonisation of Technical Requirements for Registration of Pharmaceuticals for Human Use (ICH); and with the Declaration of Helsinki—Recommendations Guiding Medical Doctors in Biomedical Research Involving Human Subjects. The current study was approved on 10 January 2022, with approval number 17.

Statistical analysis was performed using the IBM SPSS software version 26.0. Categorical variables were represented as absolute and percentage values. Student’s t-test and Mann–Whitney U-test were used for continuous and discrete variables, respectively. The χ^2^ and Fisher’s exact tests were used for the statistical analysis of proportions. The significance threshold was set at α = 0.05. The patient loss ratio (PLR) was computed to offer comparative measures of change in patients presenting for cervical cancer investigations across subpopulations. The PLR = (PP-DP)/MA, and it was determined by dividing the difference between the pre-pandemic yearly average by subpopulation (PP) and the during-pandemic yearly number of patients by subpopulation (DP) by the monthly average during the pandemic (MA). Positive PLR levels imply a drop in investigations, while negative PLR values indicate an increase. The size of the PLR value indicates the relative degree of the investigation’s decrease or growth.

## 3. Results

**Figure 1:**
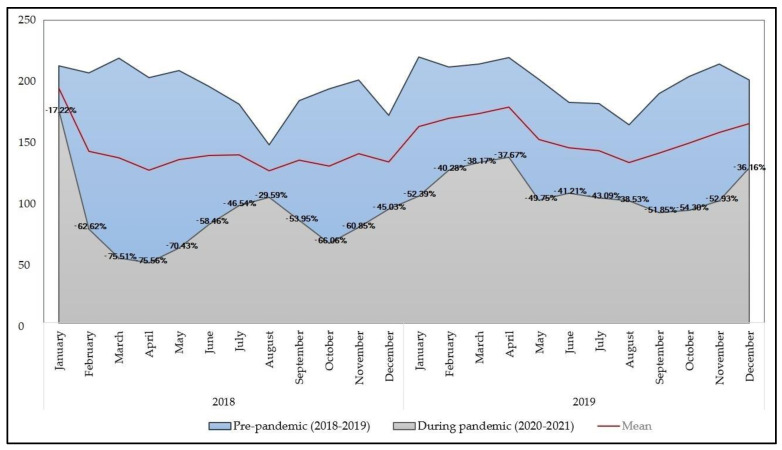
Evolution of cervical cancer screening before and during the COVID-19 pandemic.

Since the beginning of the COVID-19 pandemic in Romania in February 2020, and the subsequent lockdown measures implemented to delay the spread of SARS-CoV-2, we have seen a considerable drop in the number of cervical cancer screening tests and newly diagnosed cervical cancer patients. This decline was notably different from the pattern in the preceding two years (2018 and 2019), despite the absence of any reason to anticipate an abrupt epidemiological shift. Thus, the fundamental assumption backed by existing cancer burden data [24] is that cervical cancer typically takes between ten and twenty years to develop [25]; thus, the number of new cervical cancer cases did not naturally decrease or remained equivalent to the year previous to the start of the COVID-19 pandemic, but fewer of these new cases were effectively identified throughout the follow-up period. Figure 1 presents a detailed description of the women presenting for cervical cancer investigation at our outpatient clinic before and during the COVID-19 pandemic.

A relatively linear trend in the number of cervical cancer screening tests was observed between 2018 and 2020, with insignificant seasonal changes. However, when the pandemic started at the beginning of 2020, we observed a dramatic decrease in tests performed, dropping from −17% and −62% in January and February, respectively, down to −75% in April and May, as compared with the same months before the pandemic (Figure 1). A slight but constant uptrend continued since that period, with several months of decreased testing, consistent with the pandemic waves and government restrictions. In the last month of 2021, the numbers recovered up to −36.1% from the same pre-pandemic period, but were still significantly lower than what was considered normal before. Overall, cervical cancer investigations were significantly affected by an average percent change of 49% decrease in the volume of tests during the two years of pandemic (CI [−31.7; −68.6], *p*-value < 0.001).

**Figure 2:**
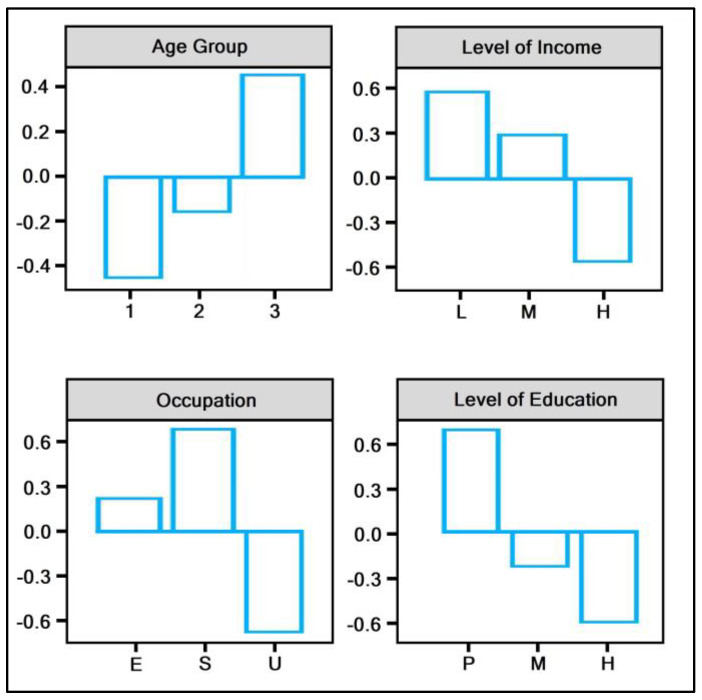
Patient loss ratio for cervical cancer investigations during the COVID-19 pandemic. Age group: 1—21–35 years; 2—35–50 years; 3—50–65 years. Level of income: L—low; M—medium; H—high. Occupation: E—employed; S—self-employed; U—unemployed. Level of Education: P—primary; M—middle; H—high.

The general characteristics and background of patients requesting cervical cancer investigations are presented in Table 1. There was a statistically significant age difference between the two study periods, both by average age of patients and by age group. The mean difference was 33.6 years before the pandemic and 32.4 years during the pandemic (*p*-value = 0.002). The majority of patients presenting for investigations before and during the pandemic was in the 21–35 years age group (53.4%, respectively, 56.8%, *p*-value = 0.003). The biggest patient loss ratio identified by age group was in the 50–65 years, as seen in Figure 2. Other significant findings were identified in the level of income, where most patients were in the medium income range (53.4% pre-pandemic vs. 51.3% during the pandemic), with the biggest patient loss ratio observed in the low-income group (from 24.3% to 19.4%, *p*-value < 0.001). There was an important decrease in the numbers of employed and self-employed patients during the COVID-19 pandemic, with the biggest PLR seen in the self-employed group (*p*-value < 0.001). Lastly, we identified a statistically significant decrease in the level of education of patients requesting cervical cancer investigations during the pandemic, where those with primary education accounted for only 7.7% of investigations compared with 13.9% before the pandemic (*p*-value < 0.001). This group was also represented by the biggest patient loss ratio (−0.6), as seen in Figure 2.

**Figure 3:**
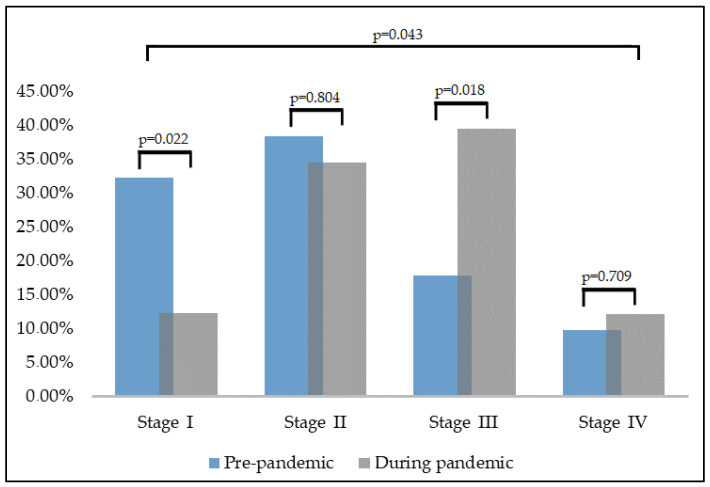
Screening outcomes for cervical cancer stages before and during the COVID-19 pandemic. The period reported as before the pandemic ranges between 2018 and 2019, while the period during the pandemic ranges between 2020 and 2021.

Overall, the number of individual tests for cervical cancer was significantly decreased during the pandemic by the percentage of Pap smears, HPV tests, and colposcopies. The percentage of individuals awaiting more than four weeks of test results was statistically significantly higher during the pandemic (5.5% vs. 14.7%, *p*-value < 0.001). The number of newly diagnosed cervical cancers was significantly lower from the baseline, with a 45% drop (CI [−31.6; −53.3], *p*-value < 0.001). Similar concerning findings were in the stage of cancers newly diagnosed, with a significant difference in stage III cancers of 21.4% more during the pandemic (*p*-value = 0.018), as shown in Figure 3. Lastly, we observed that patients newly diagnosed with cervical cancer took significantly more time to their first cancer center visit (4.1 months vs. 6.4 months, *p*-value < 0.001), and they had significantly more missed appointments than pre-pandemic times (16.1% vs. 7.6%, *p*-value < 0.001), as described in Table 2.

## 4. Discussion

Cervical cancer tests performed using Pap smears, HPV testing, and colposcopies, as well as treatment volumes decreased significantly during the first 24 months of the COVID-19 pandemic compared with the pre-pandemic period. The most significant decrease was observed within the March 2020 to December 2020 period, after which the numbers began to show a slight but consistent recovery. Consistent with the drop in cervical cancer investigation, each month between March and December 2020 saw an average of 49 percent fewer high-grade cytological abnormalities diagnosed. Although cervical screening, colposcopy, and treatment volumes had begun to rebound in area of study by January 2021, they had not yet recovered to pre-pandemic levels. As the pandemic progresses, it is expected that these services will continue to be reduced for a while due to a variety of factors, including rigorous infection prevention and control measures that limit the volume of human interactions that primary care providers can engage in. Additionally, depending on pandemic peaks, some primary care providers, such as our clinic, have varied between resuming in-person preventative care appointments and cancelling in-person visits.

Our findings indicated that individuals diagnosed with advanced-stage cervical malignancies during the pandemic compared with the same period prior to the pandemic. The percentage of late-stage cervical cancers (III–IV) rose significantly by 17%. During the pandemic era, the total number of new cervical cancer cases presenting at tertiary cancer centers was also lower. An intriguing discovery was that the percentage of cervical cancer cases detected during the pandemic dropped as a result of screening testing. Following a cancer diagnosis, the delay between the first visit to a cancer center reduced during the pandemic compared to the preceding era. Taking this into account, the primary effect of the COVID-19 pandemic on delaying cancer patients’ treatment seems to be a drop in the number of screening appointments and diagnostic procedures. The primary explanation for the reduction in the time between initial visits to a cancer center is that fewer patients were referred during the pandemic, improving access for those who were referred. Nonetheless, our cancer center is a tertiary care facility dedicated exclusively to oncologic patients. Less specialized centers that also serve non-oncologic patients may have needed a bigger shift in health care priorities to accommodate COVID-19 patients. Therefore, in such instances, the likelihood of delays in initiating cancer therapy after diagnosis cannot be ruled out.

Similar findings were confirmed by a study taking place in the US [26], where an observed reduction of more than 70% in Pap smears during the lockdown period triggered an alarm sign for oncologists. Additionally, it was reported that HPV test screening rates per 100 person-months declined by more than 80 percent among women aged 30–65 years. After the lockdown was removed, screening rates in the United States rebounded to near baseline levels, in contrast to persistent declines in our area in Romania. Other studies focused on different cancer types that are dependent on screening programs to preserve good curative results, such as breast cancer and colorectal cancer. All observations converged to the same concerning fact that the COVID-19 pandemic caused a steep decline in screening and diagnosis availability, with a negative forecast in long-term cancer outcomes [27,28,29]. As the pandemic persists, priority should be given to individuals at increased risk of acquiring cervical malignancies and pre-cancer. It is critical to continue to ensure that women obtain preventative services, such as cancer screening and adequate follow-up, in a safe and timely manner.

Although it is not yet possible to quantify the impact of COVID-19-related service reductions on cervical cancer outcomes and make long-term projections in Romania, a study conducted by Smith et al. [30] suggests that disruptions to routine cervical cancer screening in 2020 could result in an increase of 1.1–3.6 percent in cervical cancer diagnoses. The researchers assessed the effect of a variety of standardized screening interruption situations in four countries with varying cervical cancer prevention strategies. Additionally, it was expected that between 2020 and 2022, there would be a 60% increase in cervical cancers, resulting in an increase in cervical cancer mortality, morbidity, or both over the long run [31]. In addition, Gupta et al. [32] determined a 2.52% to 3.80% increase in cervical cancer related deaths with treatment delays ranging from 9 weeks to 6 months. Davies et al. [33] estimated that over the next 3 years there would be considerable rise in the number of newly diagnosed cervical cancer cases, while Kregting et al. [34] forecasted a two-fold increase in cervical cancer deaths per 100,000 individuals in 10 years. Several other studies by Matsuo et al. determined that a waiting time of between 6.1 and 9.8 weeks for cervical cancer treatment was not associated with increased risk of all-cause mortality compared to a waiting time of 6 weeks [35,36]. Additionally, in women with early-stage cervical cancer, an 8-week delay for hysterectomy may not be related with short-term disease recurrence and shorter disease-free survival [37].

Several limitations of the current study comprise the single-center design and data availability from patient records. Data collection from a single-center may not be representative for the entire Romanian population, as proportions of patient background characteristics may vary with large differences across the country. As a single-center that partially dealt with women infected with SARS-CoV-2 in the region, the significant decrease in patients that was observed during the COVID-19 pandemic might also have been influenced by physician overload and availability. Therefore, this single-center study may lack external validity. Additionally, population estimates might be different and patient migration from the area of study should also be taken into consideration. Above the aforementioned limitations, our study is the first in the region to describe in detail the variation in cervical cancer diagnosis and management, as well as providing detailed patient characteristics. It gives a first insight into the effects of the COVID-19 pandemic on this group of patients and provides valuable evidence to help prevent and further manage cervical cancer screening and newly diagnosed patients during a pandemic.

The careful monitoring of the downstream effects of COVID-19-related service interruptions on cervical pre-cancer and cancer outcomes will be important to help service providers plan and minimize adverse consequences. Based on these data, we anticipate similar future results in our area, and future priority measures for catch-up should be created that balance prospective resource limits with clinical needs.

## 5. Conclusions

The COVID-19 pandemic had a considerable detrimental influence on investigations performed to detect cervical cancer, as well as cervical cancer care in the first 24 months of the pandemic, compared to the pre-pandemic period. Although the figures are steadily improving, there is still a significant gap that might result in the missed detection of many cervical cancer cases. Not only are we missing these cases, but patients newly diagnosed with the disease are often found to have more advanced stages, and the pandemic conditions make it harder to access cancer care. We urge the implementation of further measures to close the gap between the pre- and post-pandemic periods for cervical cancer diagnosis and management.

## Figures and Tables

**Table 1:** General characteristics of patients presenting in the outpatient setting for cervical cancer investigations and treatment before and during the COVID-19 pandemic.

Variables *	Pre-Pandemic (*n* = 2281)	during Pandemic (*n* = 1340)	*p*-Value
Age, years (mean ± SD)	33.6 ± 12.1	32.4 ± 10.6	0.002
**Age group**			0.003
21–35 years	1219 (53.4%)	761 (56.8%)	
35–50 years	637 (27.9%)	388 (28.9%)	
50–65 years	425 (18.6%)	191 (14.3%)	
**Place of origin**			0.177
Rural	794 (34.8%)	437 (32.6%)	
Urban	1487 (65.2%)	903 (67.4%)	
**Level of income**			<0.001
Low	554 (24.3%)	259 (19.4%)	
Medium	1218 (53.4%)	688 (51.3%)	
High	508 (22.3%)	393 (29.3%)	
**Occupation**			<0.001
Employed	1227 (53.8%)	706 (52.7%)	
Self-employed	696 (30.5%)	352 (26.3%)	
Unemployment	358 (15.7%)	282 (21.0%)	
**Level of education**			<0.001
Primary	316 (13.9%)	103 (7.7%)	
Middle	801 (35.1%)	494 (36.9%)	
High	1164 (51.0%)	743 (55.4%)	
**Civil status**			0.393
Married	1948 (85.4%)	1136 (84.8%)	
Single	154 (6.8%)	83 (6.2%)	
Divorced/Widowed	179 (7.8%)	121 (9.0%)	
**Parity**			0.277
Nulliparous	542 (23.8%)	291 (21.7%)	
Primigravida	645 (28.3%)	404 (30.2%)	
Multiparous	1094 (47.9%)	645 (48.1%)	

* Data presented as *n* (%) unless specified differently; SD—standard deviation; pre-pandemic period—2018–2019; pandemic period—2020–2021.

**Table 2:** Medical investigations characteristics of patients presenting in the outpatient setting for cervical cancer investigations and treatment before and during the COVID-19 pandemic.

Variables *	Pre-Pandemic (*n* = 2281)	during Pandemic (*n* = 1340)	*p*-Value
**Individual tests**			
Pap smear, *n* = 3125	2017 (88.4%)	1148 (85.7%)	0.015
HPV test, *n* = 574	383 (16.8%)	191 (14.3%)	0.043
Colposcopy, *n* = 149	108 (4.7%)	41 (3.1%)	0.014
Awaiting results (>4 weeks)	126 (5.5%)	197 (14.7%)	<0.001
High-grade cytology result	258 (11.3%)	159 (11.9%)	0.613
Newly diagnosed cervical cancers, *n* = 105	72 (3.2%)	33 (2.5%)	0.229
Cancer stage **			0.043
Stage I	24 (33.4%)	4 (12.1%)	0.022
Stage II	28 (38.9%)	12 (36.4%)	0.804
Stage III	13 (18.0%)	13 (39.4%)	0.018
Stage IV	7 (9.7%)	4 (12.1%)	0.709
Interval from biopsy result to first cancer center visit, months (median [IQR])	4.1 [2–9]	6.4 [3–11]	<0.001
Missed appointments	174 (7.6%)	216 (16.1%)	<0.001
Cervical cancer-related mortality	219 (9.6%)	184 (13.7%)	0.366

* Data presented as *n* (%) unless specified differently; IQR—interquartile range; ** based on the FIGO staging system; pre-pandemic period—2018–2019; pandemic period—2020–2021.

## Data Availability

The data presented in this study are available on request from the corresponding author.
